# Arterial spin labeling perfusion MRI applications in drug-resistant epilepsy and epileptic emergency

**DOI:** 10.1186/s42494-023-00134-3

**Published:** 2023-09-28

**Authors:** Yingchun Xu, Ge Tan, Deng Chen, Jiao Liu, Zixian Zhou, Ling Liu

**Affiliations:** Department Neurology, Sichuan University, West China Hospital, Guo Xue Lane 37, Chengdu, 610041 Sichuan PR China

**Keywords:** Arterial spin labeling, Cerebral blood flow, Drug-resistant epilepsy, Epileptic emergency, Status epilepticus, SUDEP

## Abstract

Epilepsy affects all age groups and is one of the most common and disabling neurological disorders worldwide. Drug-resistant epilepsy (DRE), status epilepticus (SE), and sudden unexpected death in epilepsy (SUDEP), which are associated with considerable healthcare costs and mortality, have always been difficult to address and become the focus of clinical research. The rapid identification of seizure onset and accurate localization of epileptic foci are crucial for the treatment and prognosis of people with DRE, SE, or near-SUDEP. However, most of the conventional neuroimaging techniques for assessing cerebral blood flow of people with epilepsy are restricted by time consumption, limited resolution, and ionizing radiation. Arterial spin labeling (ASL) is a newly powerful non-contrast magnetic resonance imaging technique that enables the quantitative evaluation of brain perfusion, characterized by its unique advantages of reproducibility and easy accessibility. Recent studies have demonstrated the potential advantages of ASL for the diagnosis and evaluation of epilepsy. Therefore, in this review, we discussed the complementary value of ASL in evaluating and characterizing the basic substrates underlying refractory epilepsy and epileptic emergencies.

## Introduction

Epilepsy is one of the most common neurological disorders, with a high prevalence that can affect people of all ages, races, social classes, and geographical locations [1]. Long-term recurrent and uncontrolled seizures place patients with epilepsy under great mental stress, severely decrease their quality of life, and increase mortality rates, imposing a serious burden on their families and society [2, 3]. Drug-resistant epilepsy (DRE), status epilepticus (SE), and sudden unexpected death in epilepsy (SUDEP) remain the most challenging clinical problems that are difficult to address. To optimize management, precise localization of epileptogenic lesions plays a significant role in the diagnosis, therapeutic regimen choice, and prognosis assessment of patients with epilepsy [4].

Previous studies have demonstrated that cerebral blood flow (CBF) alterations in epileptic foci are strongly associated with the ictal and interictal periods. The ictal increase and interictal decrease in regional CBF have not been fully utilized in the investigation of seizures [5–8]. Under circumstances in which no obvious lesions are found on structural magnetic resonance imaging (MRI), interictal 18-F deoxyglucose positron emission tomography (PET) and ictal single-photon emission computed tomography (SPECT) could help to provide detailed information about CBF to assist in localization [9, 10]. However, most of the techniques mentioned above are restricted by limited resolution and ionizing radiation, and neuroimaging tools that are noninvasive and non-radiological may have wider clinical applications.

As a noninvasive non-contrast MRI technique that uses magnetically labeled water in the blood as an endogenous tracer, arterial spin labeling (ASL) has recently been reported to aid in the localization of epileptic foci. An increasing body of research has shown that ASL MRI sequences are used to provide MR-based CBF quantification [11]. This review provides a comprehensive overview of the most important discoveries regarding the recent developments in ASL applications in DRE, SE, and SUDEP.

## Principles of ASL

**Fig. 1:**
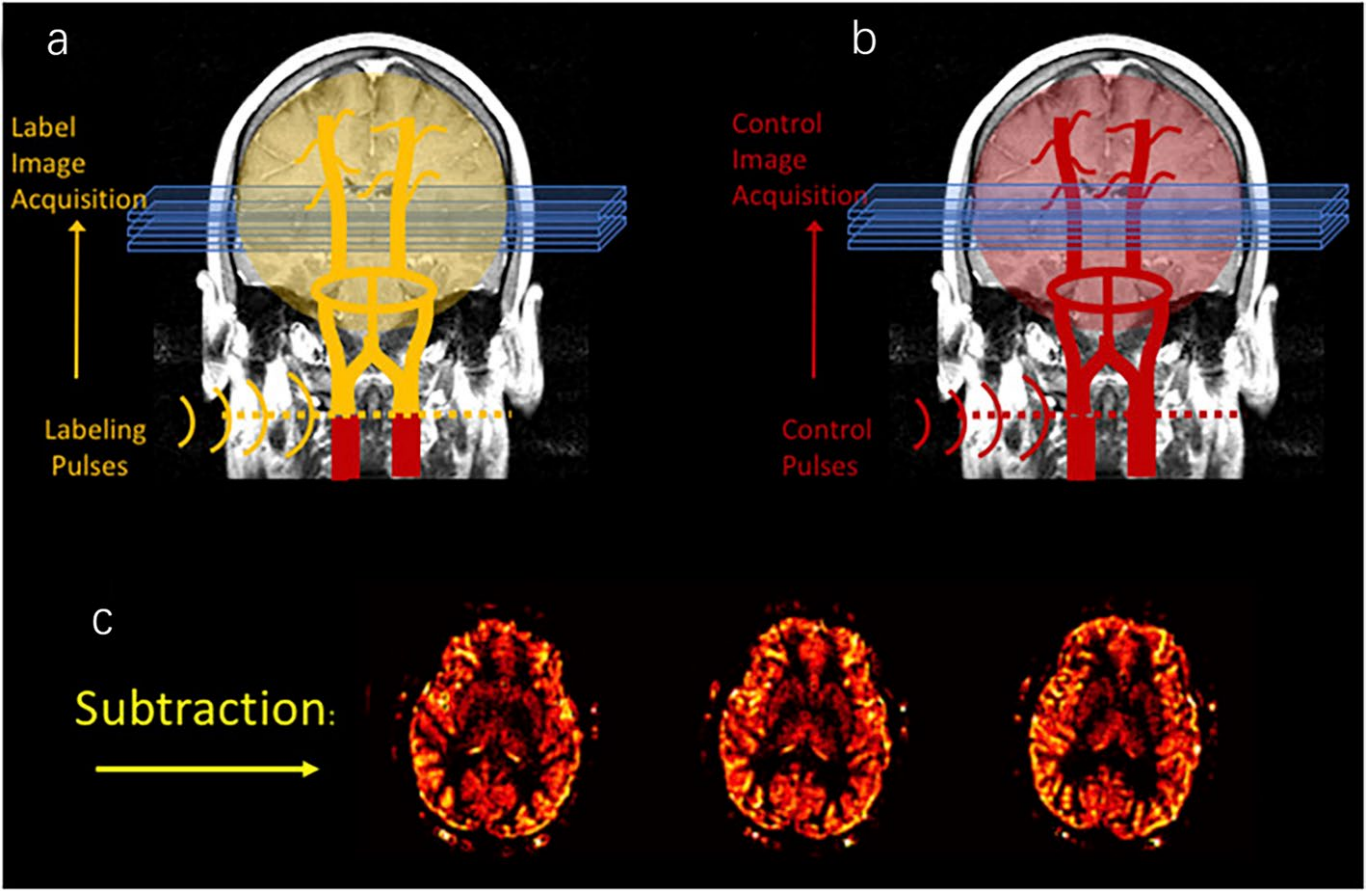
Principles of ASL. A "labeled" image and a "control" image are respectively acquired when the blood-water magnetization is inverted or not. The subtraction of these two images yields a perfusion-weighted image. Permission was granted by Hernandez-Garcia et al. (© Elsevier [14]) to reuse this figure

ASL is a MRI technique that applies radiofrequency pulses to label the water protons of arterial blood as endogenous tracer to obtain information on tissue perfusion [12]. ASL techniques are mainly utilized to produce a flow-sensitized or labeled image and a control image in which the static tissue signals are identical but the magnetization of the inflowing blood is different [13]. A labeled image and a control image are respectively acquired when the blood-water magnetization is inverted or not. And the amount of magnetization inverted and delivered to the tissue determines the signal difference between the labeled and control image. If all labeled blood reaches the imaging voxel during image acquisition, the signal difference will be proportional to CBF **(**Fig. 1**)** [11, 14].

Compared with conventional imaging modalities for CBF quantification, ASL is radiation-free, non-contrast, and reproducible, thus avoiding the risks arising from tracer or radiation exposure in younger children or patients with impaired kidney function [15, 16]. However, some important shortcomings of ASL cannot be ignored during utilization in the case of quantification error, such as sensitivity to transient time and low signal-to-noise ratio (SNR) [13]. Appropriate correction by a global scaling factor requires accurate modeling to account for multiple considerations, including labeling efficiency, flow rate, tracer delivery, magnetization decay and dispersion, water exchange, and differential relaxation of tissue compartments [17–19]. Nevertheless, in recent years, with the accessibility of 3 T MRI, the improvement of pulse sequences, and the development of multichannel receiver array coils, the disadvantages of ASL have been gradually overcome, and numerous clinical applications are being progressively performed [11].

## ASL and drug-resistant epilepsy

**Fig. 2:**
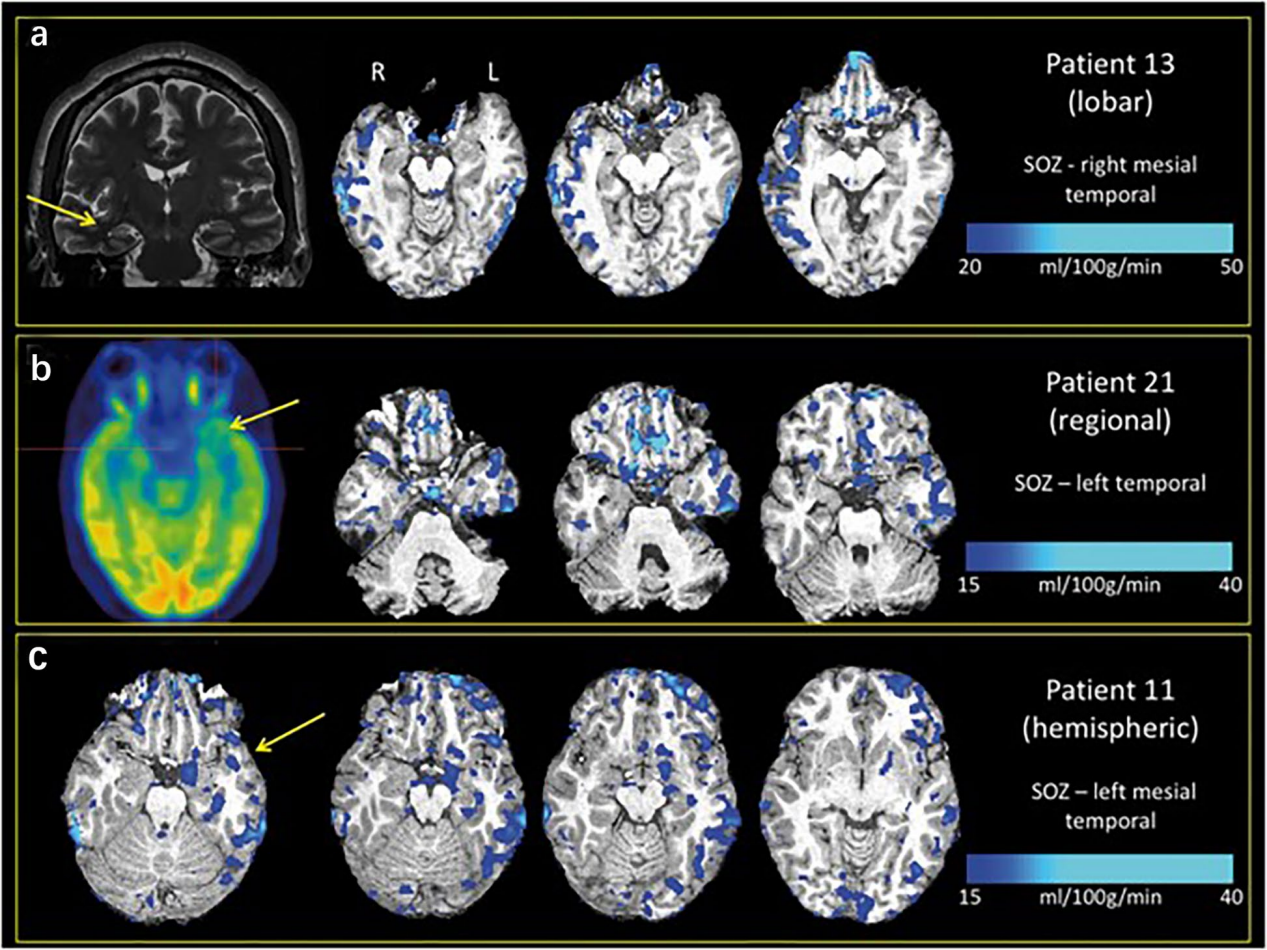
Examples of different patterns of postictal hypoperfusion seen on subtracted ASL. **a** Right mesial temporal sclerosis (arrow) on MRI and a "lobar" pattern of hypoperfusion on postictal ASL; the presumed seizure onset zone (SOZ) was in the right mesial temporal lobe. **b** Left temporal hypometabolism (arrow) on interictal PET image and a "regional" pattern of hypoperfusion on postictal ASL; the presumed SOZ was in the left temporal lobe. **c** No lesions on MRI and independent, bilateral mesial temporal SOZs based on intracranial VEEG monitoring, and a left "hemispheric" pattern of hypoperfusion on postictal ASL from a left mesial temporal seizure. Permission was granted by Gaxiola-Valdez et al. (© Oxford University Press [22]) to reuse this figure

Despite the availability of multiple antiseizure medication regimens, drug resistance is still observed in one-third of patients with epilepsy [20]. However, epilepsy surgery may represent a valuable treatment option for 10–50% of patients with pharmacoresistant epilepsy, which depends significantly on the accurate presurgical localization of the epileptogenic foci [21]. In recent years, ASL has been widely utilized in the evaluation of DRE indicated for surgery and compared with other imaging modalities. Gaxiola-Valdez et al. found that the location of hypoperfusion evaluated by ASL in 80% of patients with focal DRE was partially or fully concordant with the location of the presumed seizure onset zone determined by scalp video-electroencephalography (VEEG). Compared favorably to other neuroimaging modalities, ASL is similar or superior to structural magnetic resonance imaging (sMRI) in 71.4% of cases, ictal SPECT in 60% of cases, and interictal PET in 71% of cases (Fig. 2) [22]. Similar conclusions were also reached by Lam et al., which demonstrated ASL is a safe, non-invasive, and relatively inexpensive tool for detecting postictal hypoperfusion that may provide useful data to localize the seizure onset zone [23]. In a study conducted by Sierra-Marcos et al., 25 patients were included, and the results showed ASL had a very good concordance with FDG-PET (kappa coefficient = 0.84), good concordance with sMRI (kappa coefficient = 0.76), moderate concordance with VEEG monitoring (kappa coefficient = 0.53) and fair concordance with subtraction ictal single-photon emission computed tomography co-registered to MRI (SISCOM) (kappa coefficient = 0.28), which also suggests that ASL may be useful in confirming the location and extent of the epileptogenic zone in patients with drug-resistant neocortical epilepsy [24]. Furthermore, Khalaf et al. found that ASL significantly increased the localization specificity and positive predictive value of focal epilepsy when combined with PET, and a high concordance was found between focal FDG hypometabolism and ASL hypoperfusion, which is consistent with some other studies [25–27]. Galazzo et al. recruited 12 patients with focal DRE admitted for presurgical assessment, significant CBF changes were detected by ASL, which well matched the electrophysiological information. Good concordance between ASL and electrical source imaging (ESI) results was also demonstrated, providing further evidence that ASL can be a powerful aid in identifying epileptic activity-related CBF changes [28, 29]. In the past, patients with epilepsy with no clear lesions found by sMRI (MR negative) were typically not recommended to undergo surgery. However, with the rapid development of neuroimage techniques such as SPECT and PET, regional CBF and metabolic abnormalities in patients with epilepsy have already become crucial indicators for preoperative evaluation. In a clinical study, ten patients underwent a complete presurgical evaluation, and the presurgical FDG-PET and ASL scans were compared with the resection masks using asymmetry index (AI) maps. The results showed a better positive predictive value in six patients and sensitivity was better in four patients using ASL [30]. These results indicated that ASL is a relatively useful method for presurgical evaluation in patients with no lesions on cerebral sMRI. Given that the main benefits of ASL over PET are that it avoids radiation exposure for patients, and offers lower costs, higher availability, and better time efficiency, ASL may be used as a complementary procedure to FDG-PET or could replace FDG-PET someday [31, 32]. To better identify the preoperative evaluation value of ASL, Zheng et al. pooled the results of six studies that included 174 patients by meta-analysis and concluded that the accuracy of ASL for localizing epileptic lesions was approximately 0.88 (accuracy in ASL/all perfusion changes in ASL), further demonstrating the potential advantages of ASL for epileptic lesion localization [33]. Table 1 presents the detailed concordance between ASL and other different preoperative evaluation techniques for detecting abnormalities in patients with focal epilepsy [22–24, 31, 34].

**Table 1:** Concordance between ASL and other preoperative evaluation techniques for detecting abnormalities in patients with drug-resistant epilepsy

Author, year	Number of patients	Seizure type	Postictal perfusion alternations in ASL		Concordance between ASL and techniques				
				Scalp or intracranial VEEG	sMRI	Interictal PET	Ictal or peri-ictal SPECT	MEG	SISCOM
Gaxiola-Valdez et al., 2017 [22]	21, adult	drug-resistant focal epilepsy	15/21(71.4%)	12/15(80%)	15/21(71.4%)	10/14(71%)	9/15(60%)	NA	NA
Lam et al., 2021 [23]	25, children	focal epilepsy	17/25(68%)	12/18(66.7%)	17/20(85%)	15/20(75%)	10/16(62.5%)	6/8(75%)	NA
Sierra-Marcos et al., 2016 [24]	25, adult	drug-resistant neocortical epilepsy	15/25(60%)	15/25(60%)	20/25(80%)	15/17(88.2%)	NA	NA	6/17(35.3%)
Galazzo et al., 2016 [31]	20, adult	refractory focal epilepsy	NA	NA	All negative MRI	18/20(90%)	NA	NA	NA
Shang et al., 2018 [34]	20, adult	temporal lobe epilepsy	NA	NA	All negative MRI	14/19(73.7%)	NA	NA	NA

Abbreviations: *ASL* arterial spin labeling, *MEG* magnetoencephalography, *MRI* magnetic resonance imaging, *NA* not applicable, *PET* positron emission tomography, *sMRI* structural magnetic resonance imaging, *SISCOM* single-photon emission computed tomography co-registered to MRI, *SPECT* single-photon emission computed tomography, *VEEG* video electroencephalogram

In summary, ASL showed relatively accurate epileptic foci positioning compared with other established preoperative assessment techniques and is promising for inclusion in the routine preoperative evaluation of epilepsy [35]. A combination of several auxiliary examinations may significantly optimize the accurate localization of epileptogenic lesions.

## ASL and status epilepticus

**Fig. 3:**
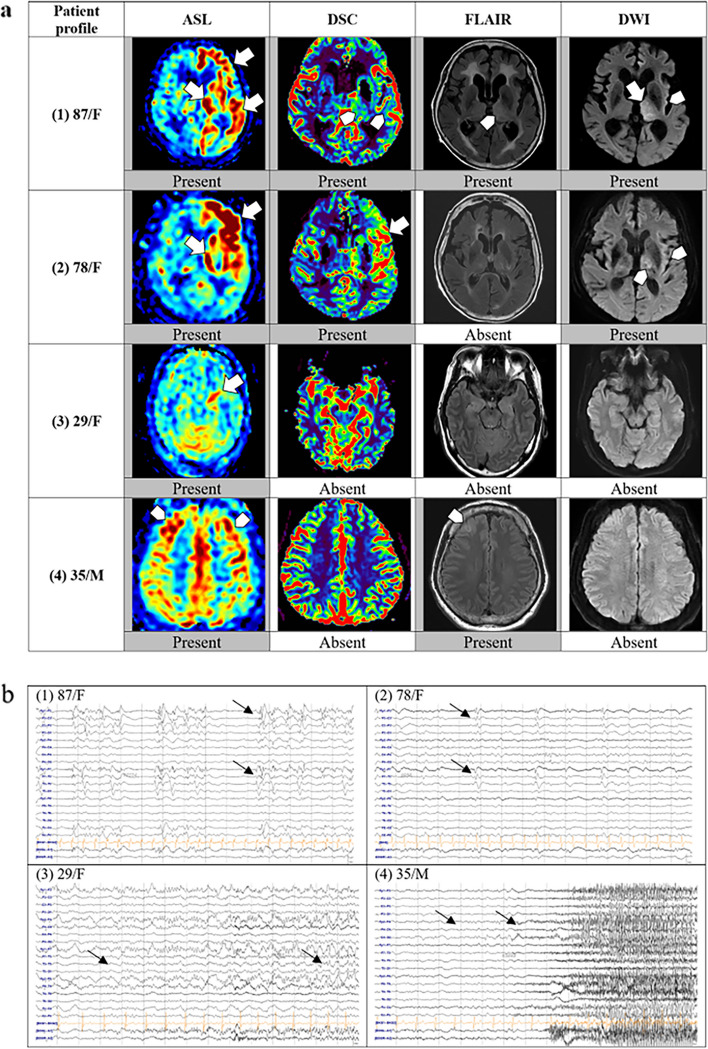
Examples of brain MRI sequences and EEGs of four representative patients with SE. **a** Brain MRI: (1) The left frontal, temporal cortices and left thalamus hyperperfusion on ASL; subtle increased perfusion in the left thalamus and lateral temporal lobe on dynamic susceptibility contrast (DSC); the left thalamus and insula changes on fluid-attenuated inversion recovery (FLAIR) and DWI. (2) Definite left frontal and temporal lobes hyperperfusion on ASL and DSC; no changes on FLAIR; subtle restrictions in the left thalamus and insula on DWI. (3) Definite left hippocampus hyperperfusion on ASL; no signal changes on DSC, FLAIR, or DWI. (4) Subtle hyperperfusion in the bilateral frontal cortices on ASL; subtle T2 hyperintensity in the left frontal cortex. **b** EEG: (1) Left frontotemporal periodic discharges. (2) Left frontotemporal periodic discharges. (3) Left temporal rhythmic delta activity with evolution indicating an electrographic seizure. (4) Right frontal theta rhythm evolving to beta activity, indicating an electrographic seizure. Permission was granted by Kim et al. (© Springer Nature [38]) to reuse this figure

**Fig. 4:**
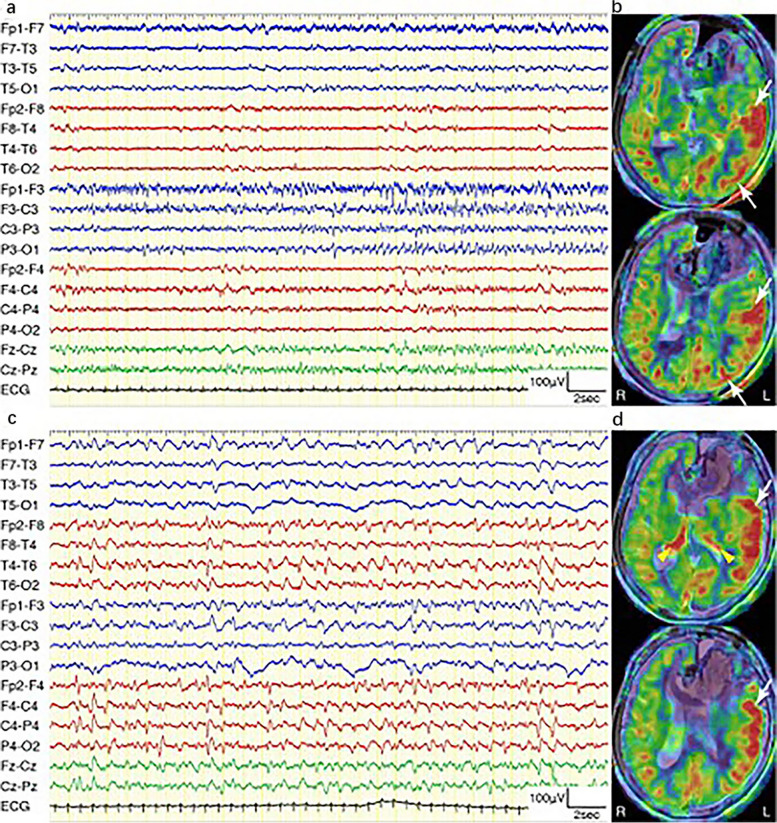
Thalamic and cerebral cortical hyperperfusion on ASL in patient with separate two NCSE episodes. **a**, **b** Episode 16; **c**, **d** Episode 17. **a** EEG showing apparent spatiotemporal evolution from the left frontal to left temporo-parietal regions. **b** ASLshowing left hemispheric cortical hyperperfusion (white arrows) without thalamic hyperperfusion. **c** EEG showing bilateral 1 Hz periodic discharges with right hemispheric predominance. **d** ASL showing bilateral thalamic hyperperfusion (yellow arrowheads) and left fronto-temporal cortical hyperperfusion (white arrows). Permission was granted by Ohtomo et al. (© Oxford University Press [43]) to reuse this figure

SE is a common neurological emergency defined as a prolonged seizure or multiple seizures with an incomplete return to baseline, with an annual incidence of 10–41 per 100,000 people, and tends to cause considerable associated healthcare costs, morbidity, and mortality [36]. The utilization of PET or SPECT during seizures can reveal an increase in local CBF in the affected area [9]; however, they are inapplicable to epileptic emergencies such as SE for their complicated and time-consuming operation and high cost, thus more convenient and rapid evaluation measures are essential. Numerous researchers have conducted significant studies on the diagnostic sensitivity and prognostic value of ASL for SE. In a study performed by Mastuura et al., the positivity rate of ASL in detecting seizure lesions in the peri-ictal phase of SE was approximately 65%, which was comparable to the sensitivity of diffusion-weighted imaging (DWI) and EEG. Additionally, ASL may detect reactive hyperperfusion associated with seizures earlier than DWI. However, two patients in the normal ASL group showed diffuse high-intensity DWI and diffuse EEG abnormalities, suggesting that ASL may not detect diffuse hyperperfusion associated with seizures [37]. In another study, the authors included 51 patients diagnosed with SE and found that ASL was more sensitive than other MR protocols or EEG in detecting refractory SE (89.5%) or estimating poor outcomes (100%), although the specificity of ASL was very low at 9.4% and 15.6%, respectively (Fig. 3) [38]. Therefore, ASL is valuable for the initial assessment of SE. Moreover, ASL has been shown to play a vital role in the longitudinal monitoring of CBF changes and follow-up of patients with SE, owing to its reproducibility and lower cost. Espinosa-Jovel et al. reported a case of autonomic SE due to limbic encephalitis in which ASL showed hyperperfusion of the hippocampus and amygdala, with subsequent multiple ASL examinations demonstrating improved results as the patient responded to treatment. During the evaluation process, ASL presented the unique advantages of feasibility and repeatability, which cannot be replaced by PET and SPECT [39]. Some patients with SE have overt convulsive activity (tonic and/or clonic) making it easy to diagnose clinically [40]. Others lacking such overt convulsive activity and presenting in protean ways are collectively referred to as non-convulsive status epilepticus (NCSE), which is difficult to diagnose; however, there is an impairment of consciousness and neuronal injury associated with ongoing seizure activity on EEG [41]. Therefore, early recognition and appropriate treatment are essential. Current research has also reported the use of ASL in NCSE. Yamamoto et al. reported two cases of elderly patients with NCSE presenting primarily with bradylalia in acute settings who were diagnosed using emergent ASL perfusion MRI [42]. This further identified the advan tage of ASL imaging in providing valuable information regarding cerebral perfusion status in emergency settings. Ohtomo et al. identified 27 patients who underwent both ASL and EEG within 24 h of suspected NCSE by comparing hyperperfusion on ASL with periodic/rhythmic discharges on EEG and concluded that thalamocortical hyperperfusion could be a new biomarker of NCSE in critically ill patients (Fig. 4) [43].

Generally, ASL has high sensitivity for the diagnosis of SE and is suitable for adjunctive diagnosis in SE emergency settings, which require immediate decisions on further treatment and is convenient for follow-up during and after treatment.

## ASL and sudden unexpected death in epilepsy

**Fig. 5:**
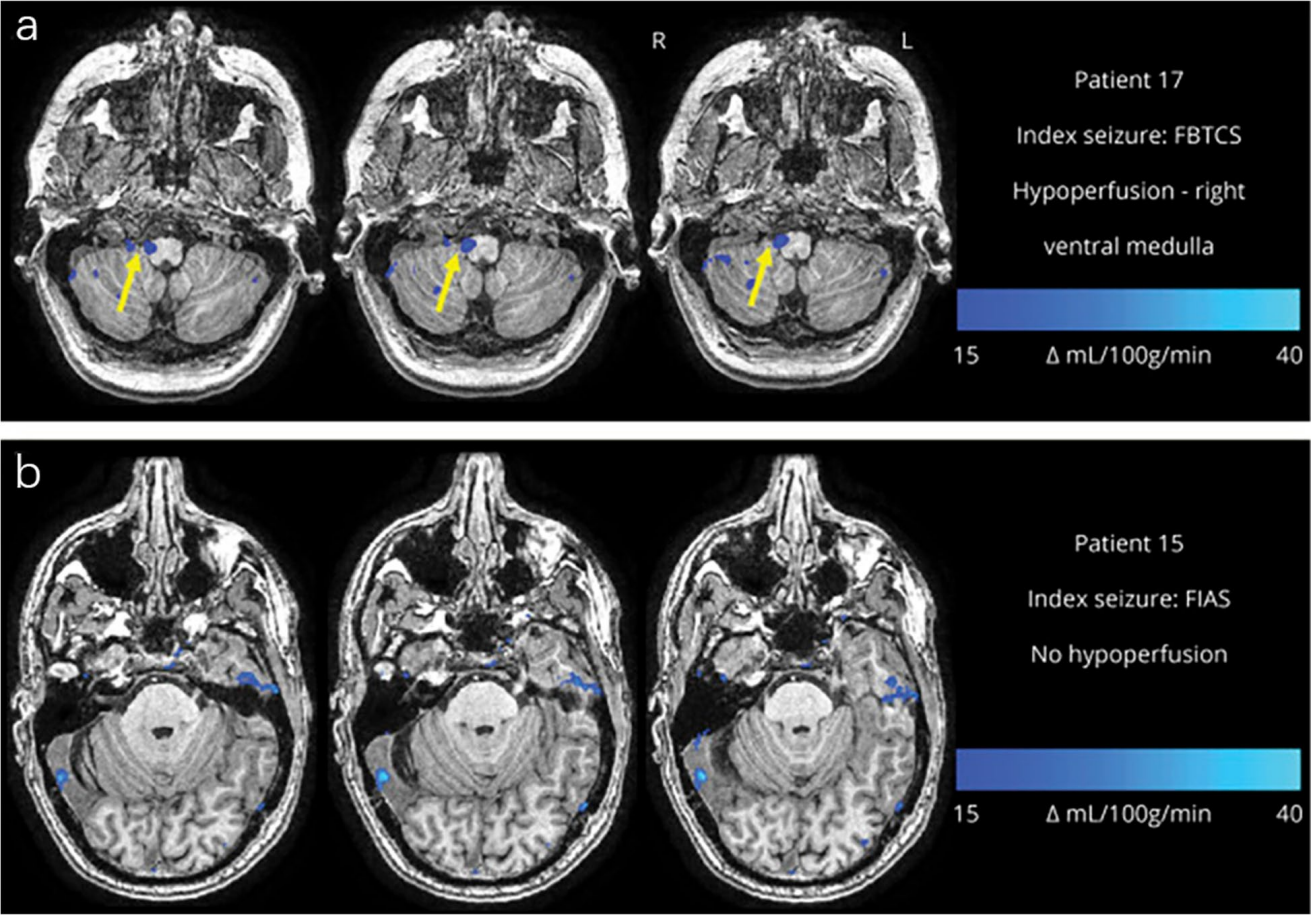
Examples of different patterns of postictal hypoperfusion in brainstem respiratory centers. **a** Image from a patient with monthly focal to bilateral tonic-clonic seizures (FBTCS). Subtraction cerebral blood flow (CBF) map (baseline-postictal) showed hypoperfusion > Δ15 CBF units in the right ventral medulla (yellow arrow) across 3 axial slices. **b** Image from a patient with infrequent FBTCS (< 1 per year). Subtraction CBF map (baseline-postictal) showed no significant hypoperfusion in any of the brainstem respiratory center regions of interest. Permission was granted by Liu et al. (© Wolters Kluwer Health [45]) to reuse this figure

SUDEP is an important cause of direct epilepsy-related death and premature death in patients with refractory epilepsy [44]. Currently, there are few relative studies on ASL and SUDEP. ASL is now primarily used to explore the pathogenesis of SUDEP in scientific research instead of clinical applications. Liu et al. used ASL to detect brainstem perfusion after seizures in patients with focal epilepsy and found that patients with bilateral tonic–clonic seizures were more likely to have brainstem respiratory center hypoperfusion, which further demonstrates the increased risk of SUDEP in patients whose seizures manifest as bilateral tonic–clonic seizures (Fig. 5) [45]. Given the reproducibility, and low cost of ASL, an increasing number of studies will include it as an evaluation tool for exploring its mechanism or identifying SUDEP in the future.

## Conclusions

ASL is a newly developed MRI technique to measure perfusion that has mostly been used in research, and recently, the clinical application of ASL as a neuroimaging technique that can measure CBF non-invasively has gained increasing attention in the field of epilepsy. Providing accurate information about postictal brain perfusion that is most concordant with other evaluation techniques, such as SPECT and PET, may also serve as a reliable tool to identify seizure onset zones before surgery [46]. Moreover, in the case of epileptic emergencies such as SE or near-SUDEP, ASL can also take advantage of reproducibility, easy accessibility, and non-invasiveness to provide relatively accurate information related to CBF to aid in diagnosis. Undoubtedly, with the fast development of magnetic resonance equipment, ASL is promising to play an increasingly important role in the study of epilepsy mechanisms and clinical applications, and could be a routine component of multi-modality imaging.

## Data Availability

All data generated or analysed during this study are included in this published article.

## Abbreviations

ASL: Arterial spin labeling
AI: Asymmetry index
CBF: Cerebral blood flow
DRE: Drug-resistant epilepsy
DSC: Dynamic susceptibility contrast
DWI: Diffusion-weighted imaging
ESI: Electrical source imaging
FIAS: Focal impaired awareness seizure
FLAIR: Fluid-attenuated inversion recovery
MEG: Magnetoencephalography
MRI: Magnetic resonance imaging
NA: Not applicable
NCSE: Non-convulsive status epilepticus
PET: Positron emission tomography
SE: Status epilepticus
SISCOM: Single-photon emission computed tomography co-registered to MRI
SNR: Signal-to-noise ratio
SOZ: Seizure onset zone
SPECT: Single-photon emission computed tomography
SUDEP: Sudden unexpected death in epilepsy
VEEG: Video electroencephalogram
